# A Brush with Danger

**DOI:** 10.5811/cpcem.2018.8.39195

**Published:** 2018-09-05

**Authors:** Alexander W. Hirsch, Erica Y. Popovsky, Lise E. Nigrovic, Michele M. Burns

**Affiliations:** Boston Children’s Hospital, Harvard Medical School, Division of Emergency Medicine, Boston, Massachusetts

## CASE PRESENTATION

A five-year-old male presented to the emergency department (ED) after falling off a stool while brushing his teeth, resulting in a penetrating oropharyngeal injury. The toothbrush was impaled deeply into the left lateral soft palate with only the handle of the toothbrush visible protruding from the mouth. The patient was hemodynamically stable and was comfortable in the arms of his mother after a single dose of fentanyl. A computed tomography angiography (CTA) with three-dimensional reconstructions was performed without sedation, which showed the tip of the toothbrush terminating adjacent to the alveolar and pterygoid branches of the external carotid artery, but with no visible injury to these vessels (Image). Associated subcutaneous emphysema and muscle edema were also noted. The otorhinolaryngology service was consulted, and the toothbrush was successfully removed in the operating room without complication.

## DISCUSSION

Although the distance from the tonsillar fossa to the internal carotid artery is approximately only 25 millimeters,^1^ most children with penetrating oropharyngeal trauma can be managed expectantly. Rarely, these injuries can damage the internal carotid artery leading to thrombosis or dissection of the affected vessel risking a cerebrovascular accident.^2^,^3^ In a previous single-center cohort of children with penetrating palate trauma, the risk of stroke was 0% (95% confidence interval 0–2.5%).^4^ Although most patients with penetrating palate injuries do not require imaging, CTA is the first-line imaging modality for the ED clinicians with a sensitivity between 90–100% for vascular injury when compared to angiography.^5^

**CPC-EM Capsule**

What do we already know about this clinical entity?

Children with penetrating oropharyngeal trauma are at risk for vascular injury, although vascular complications are rare.

What is the major impact of the image(s)?

The image demonstrates the proximity of the vasculature to the soft palate and highlights the potential for vascular injury.

How might this improve emergency medicine practice?

Most penetrating oropharyngeal injuries do not require imaging, but if the exam or mechanism is concerning, computed tomography angiography should be the first-line imaging modality.

Documented patient informed consent and/or Institutional Review Board approval has been obtained and filed for publication of this case report.

## Figures and Tables

**Image:**
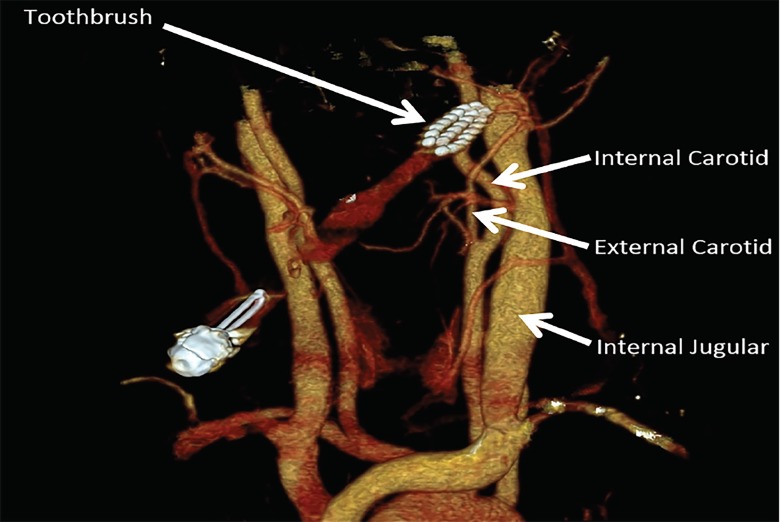
Tip of the toothbrush terminating adjacent to branches of the external carotid artery, just anterior and lateral to the left internal carotid artery and adjacent to the internal jugular vein.
